# Simulation of the Influence of the Radial Graded Porosity Distribution on Elastic Modulus of γ/β Phase Ti-Based Alloy Foams for Bone Implant

**DOI:** 10.3390/ma16237320

**Published:** 2023-11-24

**Authors:** Claudio Aguilar, Ismelí Alfonso, Daniel González, Edgar Pio, Guilherme Oliveira Neves, Flavio De Barbieri, Mamie Sancy, Lisa Muñoz

**Affiliations:** 1 Departamento de Ingeniería Metalúrgica y Materiales, Universidad Técnica Federico Santa María, Valparaíso 2390123, Chile; edgar.pio@usach.cl (E.P.); f.debarbieri@usm.cl (F.D.B.); 2 Instituto de Investigaciones en Materiales, Unidad Morelia, Universidad Nacional Autónoma de México, Campus Morelia UNAM, Morelia 58190, Mexico; ialfonso@iim.unam.mx; 3 Departamento de Metalurgia, Universidad de Atacama, Copiapó 1532463, Chile; daniel.gonzalez@usm.cl; 4 Departamento de Ingeniería Mecánica, Universidad del Biobío, Concepción 4081112, Chile; gneves@ubiobio.cl; 5 Escuela de Construcción Civil, Pontificia Universidad Católica de Chile, Santiago 7820436, Chile; mamiesancy@gmail.com; 6 Instituto de Química, Facultad de Ciencias, Pontificia Universidad Católica de Valparaíso, Valparaíso 2373223, Chile

**Keywords:** foam, Ti-based alloys, simulation, elastic modulus, radial graded porosity

## Abstract

This research aims to examine how a radial graded porosity distribution affects the elastic modulus by conducting simulations on Ti-based alloy foams with face-centered cubic and body-centered cubic crystal structures. Four types of foams were analyzed; commercially pure-Ti, Ti-13Ta-6Mn (TTM), Ti-13Ta-(TT) and Ti-13Ta-6Sn (TTS), (all in at.%). Four radial graded porosity distribution configurations were modeled and simulated using the finite element analysis (FEA). The radial graded porosity distribution configurations were generated using a Material Designer (Ansys) with a pore range of 200 to 600 μm. These radial graded porosity distributions had average porosity values of 0, 20, 30 and 40%. The consolidated samples that were obtained through a powder metallurgy technique in two step samples were synthesized using a powder metallurgy technique, with the elastic moduli values of the aforementioned Ti based alloys being measured by ultrasound using ~110, ~69, ~61 and ~65 GPa, respectively. The results showed that the modulus decreased as a function of porosity level in all simulated materials. The TTM, TT and TTS foams, with average porosities of 20, 30 and 40%, exhibited an modulus smaller than 30 GPa, which is a requirement to be used as a biomaterial in human bones. The TT foams showed the lowest modulus when compared to the other foams. Finally, certain theoretical models were used to obtain the modulus, the best being; the Gibson–Ashby model (α = 1 and *n* = 2.5) for the cp-Ti foams and Knudsen–Spriggs model (*b* = 3.06) for the TTM, TT and TTS foams.

## 1. Introduction

Advancements in science and technology have had a profound impact on society, greatly improving the quality of life for millions of people. As a result, the average life expectancy has been steadily increasing [1]. Nations characterized by advanced economies and societies enjoy life expectancies of more than 75 years, with certain countries surpassing 83 years. Japan leads the world with an average life expectancy of 85 years, while Africa lags behind with an average of around 63 years [2]. Individuals over the age of 40 face a higher risk of developing degenerative skeletal disorders resulting from the cumulative effects of wear and tear attributed to contemporary ways of living [3]. One potential way to address these issues is through the use of biomaterials as joint replacements [4,5]. However, biomaterials must meet rigorous specifications, including possessing mechanical, corrosion, osseointegration, processability, affordability, biocompatibility and durability properties without requiring additional surgeries [4,6]. Biomaterials can be categorized as ceramics, polymers, metals, or composites, with metallic materials preferred for load-bearing orthopedic implants [7]. However, only a restricted selection of materials satisfy the rigorous criteria. In this context, titanium alloys are particularly favored due to their optimal combination of properties [8,9] and are frequently utilized as replacements for jointed bones [10]. Nevertheless, titanium alloys demonstrate a significant disparity in elastic modulus (E) when compared to human bones (both trabecular and cortical), resulting in the stress-shielding phenomenon [11,12]. Pure titanium has an elastic modulus of 110 GPa [6], while titanium alloys range between 48 and 100 GPa [3], and human bones fall within the range of 1 to 30 GPa [13]. This mismatch between the E values of titanium alloys and human bones produces bone resorption, ultimately resulting in implant loosening. Therefore, titanium alloys with a lower value of the elastic modulus must be obtained to resolve the stress-shielding problem.

To address this problem, there are three approaches to lowering the Young’s modulus of Ti-based alloys. The first involves developing composite materials, which require further research to determine their properties and performance, particularly in load-bearing conditions [14]. The second approach involves altering the character of atomic bonds through alloys. This method allows Ti alloys to be obtained with a Young’s modulus ranging from 48 to 112 GPa [3]. Lastly, Ti foams (based in alloys) offer a lower E by introducing porosity into the material, allowing for customization to meet the requirements of human bones [4,13,14,15]. The last two approaches are traditionally used to obtain materials subjected to load-bearing conditions. Foams of Ti alloys have been reported with different porosity values. For example, for 50% porosity, Ti-13Ta-6Sn exhibited an E value of 4 GPa, while Ti-34Nb-29Ta-6Mn showed an E value of 33 GPa [4,16]. Additionally, porosities ranging from 50% to 85% resulted in E values varying from 24 to 5 GPa for Ti-4Al [17]. In the case of Ti-5W with 32% porosity, the E value measured was 23 GPa [18]. For foams based on Ti-6Al-4V alloys, Young’s modulus values ranging from 2.0 to 3.2 GPa were measured for porosities between 81% and 21% [19,20].

Several methods are utilized for the synthesis of metallic foams [21], including the powder metallurgy method [4], the casting method [22], the direct foaming method [23], the hollow sphere method [13] and additive manufacturing [24]. Each of these techniques yields metallic foams with varying characteristics and properties. The technique of 3D additive manufacturing is at the forefront of developing composite devices for bone implants, utilizing Ti-based alloys for their superior mechanical properties and biocompatibility [25]. These implants are customized to individual patient’s anatomy, ensuring an enhanced fit and reduced risk of complications. Integrated functionalities in these Ti-based implants encompass porous structures [26] that promote bone in-growth, surface modifications for superior osseointegration, and the potential incorporation of localized drug delivery systems [27]. Continuous advancements in this field are exploring the integration of smart technologies into implants, making significant strides in orthopedic medicine with the use of Ti-based alloys [28].

As seen, porosity has an effect on the Young’s modulus; therefore, porosity should be designed to achieve the desired modulus value. For this purpose, as a first approach, theoretical models can be used. Some semi-empirical models have been used to compute the modulus, such as the models provided by Gibson and Ashby [29], Pabst and Gregorova [30], Spriggs [31], Phani and Niyogi [32], Warren and Kraynik [33], Knudsen [34], Nielsen [35], and Zhu [36]. In those theoretical models, porosity is often considered as a key factor in calculating the E. However, in certain cases, other parameters are required, such as pore stacking, pore distribution geometry, pore size and shape, critical porosity, and connectivity, shape factor and the overall porous structure, among others. The primary limitations of the equations to determine Young’s modulus are as follows: (i) an average porosity is used, (ii) a uniform distribution of porosity throughout the material is considered, (iii) the majority of pores are considered to have a spherical shape without taking into account the shape factor, and (iv) some models were originally developed for non-metallic materials and may not be directly applicable to metals. While these models can be utilized as a first approach, it is advisable to employ them only when all synthesized parameters are meticulously controlled and measured. Consequently, extensive experimental work is essential for measuring and characterizing the parameters necessary for the accurate application of these theoretical models. In typical metallic foams produced through traditional methods like space-holder, sintered powders, or replication, achieving precise control over the porous structures is challenging [37].

Therefore, finite element analysis (FEA) proves to be a powerful tool for calculating the E values. The accuracy of E values’ calculation relies on the type of the porosity parameters employed [38]. The complexity increases when dealing with materials that exhibit graded porosity in certain dimensions, such as radial, angular, or height, especially when considering the cylindrical coordinate system. In the literature, only a limited number of studies address foams with graded porosity, and even fewer focus on foams made from Ti-based alloys. All of them solely present experimental results and do not conduct E simulations using FEA [39,40,41,42,43].

Pure Ti and its alloys have two equilibrium crystal structures, having a hexagonal close-packed (hcp) structure when at lower temperatures, and transforming into a body-centered cubic (bcc) structure at higher temperatures, also known as α-phase and β-phase, respectively. The transformation temperature of α-phase to β-phase (called β-transus) can be changed by modifying the temperature, pressure and chemical composition [6], i.e., for pure Ti at 1 atm, the β-transus would be 882 ± 2 °C. Commonly for Ti alloys, the β-transus is habitually changed by adding alloying elements [6,44]. Ti-based alloys that have a β-phase are preferred over others to be used as biomaterials since they have a lower Young’s modulus than that of Ti-based alloys with an α-phase [45]. The typical elements used to reduce the β-transus, and stabilize β-phase at room temperature, are Mo, V, Ta, Zr and Nb (β-isomorphous) [46] and Co, Fe, Cr, Ni, Mn, Cu, Si, H (β-eutectoid) [47,48]. Additionally, a metastable face cubic-centered (fcc) crystalline structure (γ-phase) has been observed in pure Ti and some Ti alloys [49,50,51,52,53,54,55,56,57]. Ti-based alloys with a γ-phase have been obtained using different methods, such as explosive cladding [51], rolling at room temperature for pure Ti [58], water quenching of compact Ti [59], and high-energy milling [52,60]. Common features of synthesis processes that have been obtained at γ-phase are: (i) nanocrystalline grain size and (ii) high deformation. Despite that, the formation of the γ-phase has been reported in several Ti alloys, while there is no information about foams with this crystal structure. This may be due to the following reasons; (i) Ti-based alloy foam manufacturing methods use high temperatures, and since γ-phase is metastable, it disappears when high temperatures are applied and (ii) it is very complex to synthesize Ti alloy foams at lower temperatures. It is feasible to retain the γ-phase at lower temperatures with a very high load of compaction being necessary (>1.0 GPa) when foams are synthesized using a powder metallurgy route. Therefore, when applying a very high load, the space-holder particles collapse, thus fracturing and producing an irregular pore structure with sharp-edged pores, along with pore walls that have different thicknesses. These characteristics do not favor foams being used as biomaterials. However, synthesizing Ti-based alloy foams with a fcc structure remains a concern since other possibilities could perhaps be opened to new biomaterials. In this context, exploring the effect that graded porosity has on the E of Ti alloy foams with a fcc structure through modelling and simulation makes further experimental study reasonable and interesting.

Therefore, the novelty of this work is to simulate and predict by FEA the Young’s modulus of three Ti alloy foams with a γ/β phase ratio with a distribution of porosity. It should be noted that γ-Ti alloy foams were not obtained due to the complexity of the process of synthesis. The foam models were created using computer-aided design (CAD) software, which incorporated three radial zones with varying porosity levels and the simulations were conducted in compression way. The Young’s modulus of three alloys (consolidated samples) was obtained experimentally by ultrasound, (i) Ti-13Ta-6Mn (at.%), (ii) Ti-13Ta (at.%) and (iii) Ti-13Ta-6Sn (at.%).

## 2. Materials and Methods

### 2.1. Metallurgical Design of Alloys

In this work, three γ/β Ti-based alloys were chosen according to the following metallurgical criteria:

(a) **Microstructure:** The Ti-based alloys must be composed with a high γ/β phase ratio.
(b) **Synthesis method**: As the γ-phase is a metastable phase, a non-equilibrium method was used to synthesize it. In this context, mechanical alloying exhibited some advantages: (i) it is a simple and versatile technique, (ii) it has very large departures from equilibrium, with the maximum departure from equilibrium being ~30 kJ/mol, and (iii) there is a possibility of obtaining materials with nanocrystalline grain size and high deformation, which are two of the characteristics required to synthesize Ti-based alloy with γ-phase, through mechanical alloying.
(c) **Ti-based alloys**: Three Ti-based alloys were chosen according to previous studies completed by our group and reported by other works where the γ-phase was obtained by mechanical alloying. The alloys selected were Ti-13Ta-6Mn (TTM), Ti-13Ta-(TT) and Ti-13Ta-6Sn (TTS) (all in at.%).
(d) **Consolidated samples**: Only consolidated samples were obtained since synthesizing foams with α γ/β-phase ratio is very complex when using traditional processes.

### 2.2. Consolidated Sample Production

Consolidated samples were obtained through a powder metallurgy technique in two steps; mechanical alloying and hot-pressing. Grade IV Ti powders (<149 µm; NOAH Technologies San Antonio, TX, USA), Ta powders (99.9% purity, -325 mesh; NOAH Technologies), Mn powders (99.9% purity, <325 mesh; NOAH Technologies) and Sn powders (99.8% purity, <100 mesh; NOAH Technologies) were used to synthesize the alloy in a planetary mill (Retsch PM400). Three Ti-based alloys were mechanically alloyed, Ti-13Ta-6Mn (TTM), Ti-13Ta-(TT) and Ti-13Ta-6Sn (TTS), (all in at.%). The powders were milled at 100 h under a protective ultra-pure Ar atmosphere to prevent oxidation. Then, 30 min/30 min on/off cycles were used to prevent the milling temperature from increasing. A jar (250 mL) and balls (balls of 5 and 10 mm in diameter) of Yttrium-stabilized Zirconia (YSZ), with a ball-to-powder weight ratio of 10:1, were used, with 2 wt.% of stearic acid being used as a process control agent. The milled powders were compacted into a hardened steel die with a diameter of 8 mm. Cylindrical samples of 8 mm in diameter and 8 mm in height were synthesized. Consolidated samples were obtained by applying hot-pressing with a thermal cycle consisting of three steps; (a) 200 °C for 5 min, (b) 500 °C for 25 min and (iii) cooling up to room temperature. The heating rate was 10 °C/min, with the heating treatment being performed under an ultra-pure Ar flow of 1 L/min.

### 2.3. Sample Characterization

The Young’s modulus was measured using an ultrasonic process and following the ASTM D2845-08 standard [61]. The Young’s modulus was obtained using an ultrasonic piezoelectric transducer contact Olympus, V156 and V110 transducers by using shear and longitudinal waves that were generated by Agilent 33220A equipment and subsequently amplified by NF BA4850 equipment. Both emission and reception signals were acquired by the Tektronix TDS2012 oscilloscope, whose temporal sensitivity was 1 ns. The X-ray diffraction (XRD) powder patterns of the alloys were recorded on a multi-purpose powder diffractometer STOE STADI MP equipped in transmission geometry, using a pure Cu Kα1-radiation source (λ = 1.54056 Å, curved Germanium (111) monochromator Johann-type, 40 kV, 30 mA) with a DECTRIS MYTHEN 1K detector. The XRD patterns were obtained by scanning from 2θ between 25° and 90° with a step size of 0.12° (2θ) and holding time of 10 s per step. The X-ray diffraction patterns were indexed using the PDF-2 database and the Crystallography Open Database (COD). The microstructural characterizations were constructed using the Rietveld Method and the software, Materials Analysis Using Diffraction (MAUD) [62,63]. The LaB6 (*a* = 4.1565 (1) Å) was used as an external standard for determining instrumental broadening [64]. To account for the microstructure of the phases, the profile fitting was performed by considering the Delf line broadening model [65,66] and an isotropic size-strain model implemented in MAUD.

## 3. Modelling and Simulation

### 3.1. RVE-FEM Method

**Figure 1:**
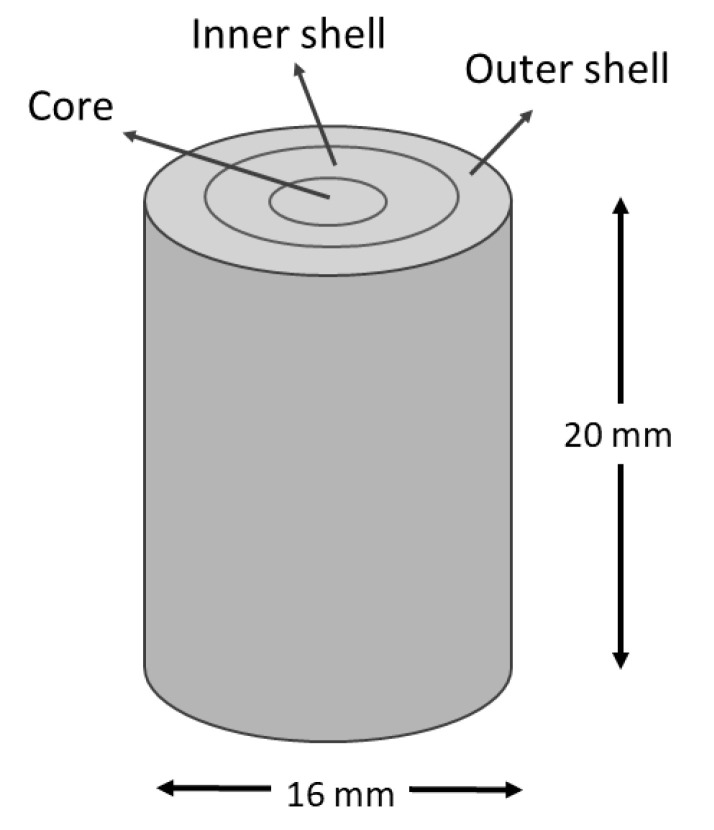
A radial graded porosity distribution scheme in a cylindrical foam.

Simulations were constructed considering the radial graded porosity distribution in cylindrical foams. The radial graded porosity distribution configurations consisted of three pore zones labeled as; core, inner shell, and outer shell, as can be seen in Figure 1. The geometrical dimensions of cylindrical foams were high (h = 20 mm) and diameter (ϕ = 16 mm). Four radial graded porosity distribution configurations (labeled as pc-1, pc-2, pc-3 and pc-4) were simulated according to data provided in Table 1. The right column gives the average porosity level for each radial graded porosity configuration. The influence of radial graded porosity distribution on Young’s modulus was simulated for four materials: (i) commercially pure (cp)-Ti, (ii) Ti-13Ta-6Mn (TTM), (iii) Ti-13Ta-(TT) and (iv) Ti-13Ta-6Sn (TTS).

**Figure 2:**
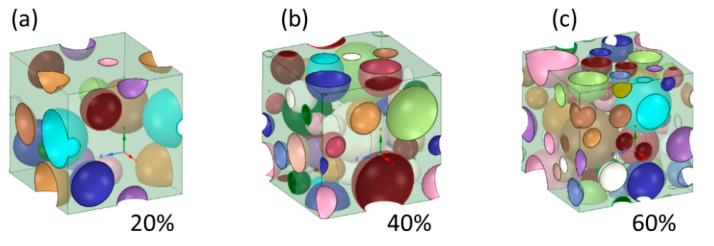
Schematic 3D foam cell model generated by Material Designer (**a**) 20%, (**b**) 40%, (**c**) 60%.

Mechanical computational simulations used to determine the Young’s constants of different metallic foams were achieved by implementing an Ansys v 19.3 software called Materials Designer (MD) module. The software has the particularity of simulating complicated microstructures involving different scale lengths, as in the case of a porous structure. The software assumes that the material is a representative structure at the microscale level. The representative volume element of this structure (RVE) is much smaller than the macroscopic volume that represents the real experimentally measured mechanical properties [67]. The material Designer Module builds models with random uniform pore size distribution ranging between 100 and 500 (µm) using spheres that are randomly distributed with no intersection between them (Figure 2a–c). The MD module does not reflect the intrinsic anisotropies of the foam creation process.

**Figure 3:**
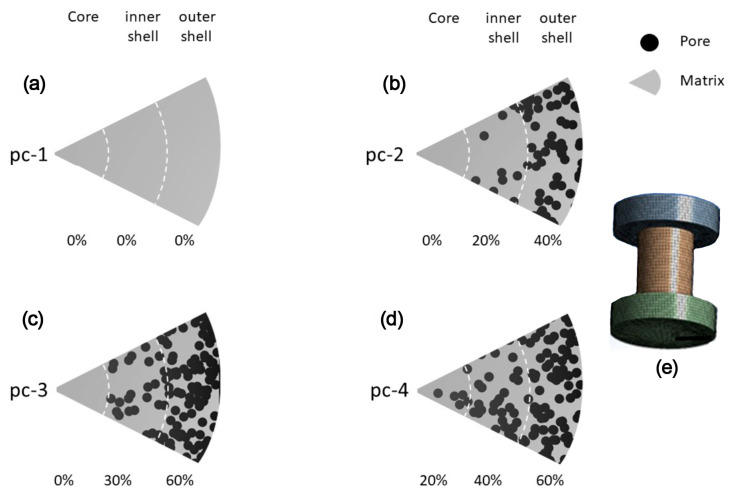
Configuration scheme of the radial graded porosity distributions, (**a**) pc-1, (**b**) pc-2, (**c**) pc-3, (**d**) pc-4 and (**e**) the simulated cylindrical specimen foam scheme.

**Figure 4:**
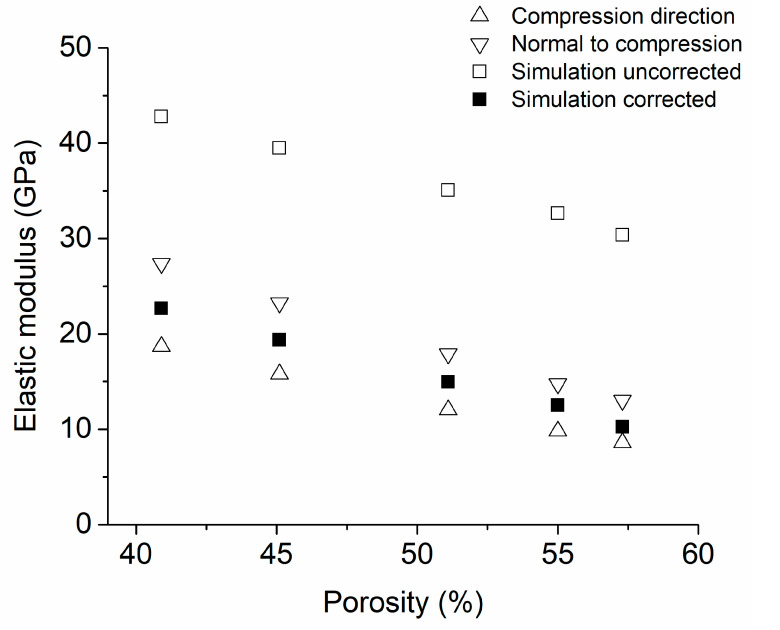
Effect of the correction factor (b) in cp-Ti foam simulations.

To validate the numerical model, computational simulations of cp-Ti foams were fabricated using the Young’s parameters listed in Table 2. The porosities used were 40, 45, 50, 55 and 60 *v*/*v*% with a uniform pore size distribution being between 100 and 500 µm in the same way that was used by Imwinkelried [47] and Tanwongwan [48]. The MD module generates a unit cell with pore distribution which is used by a structural ANSYS to generate samples with a radial graded porosity distribution configuration (Figure 3). A correction factor (β) must be introduced due to the Young’s modulus values simulated (open square) being higher than values reported in the literature concerning the compression direction (upper open triangle), and normal with the compression direction (down open triangle) for cp-Ti foams (Figure 4) [68]. This is due to the fact that the software does not capture the anisotropy behavior of the cp-Ti foams. The best fitted correction factor was −0.183, as well as the corrected Young’s modulus values being obtained (filled square) between the values of both directions, compression direction (upper open triangle) and normal to compression direction (down open triangle) (Figure 4).

After the method was validated for cp-Ti foams, the Young’s modulus for the three Ti-based alloy foams were simulated according to configurations given in Table 1 and Figure 1. The conditions that were used consist of (i) the Young’s modulus for the consolidated Ti-based alloys were experimentally measured and given in Table 2, (ii) cylindrical specimens with a diameter of 16 mm and height of 20 mm were simulated in a compression test, and (iii) to determine the Young’s modulus, the engineering stress and strain were simulated with the Young’s modulus, then calculated from Hooke’s law. The strain rate was fixed at $\overset{\cdot }{\varepsilon }$ = 0.005 s^−1^ with a friction coefficient of 0.5 between the crosshead and the specimen. The parameters used were based on the work of Tanwongwan and Carmai [69] with a mesh quality of 0.97 being achieved.

### 3.2. Simplified Theoretical Model to Estimate Young’s Modulus

As complementary work, the Young’s modulus obtained by simulations were compared with some theoretical models found within the literature. Those models estimated the Young’s modulus of foams as a function of porosity or density. Gibson and Ashby [29] proposed a model where the Young’s modulus was influenced by the relative density of foam along with two parameters (α and n), Equation (1), where ρ and $\rho_{0}$ were considered the foam and consolidated material density, respectively. With this, α varies between 0.1 and 4 and n value between 1.8 and 2.2. Xiong et al. [7] measured the mechanical properties of Ti foams and determined that α = 1 and n = 2 in Equation (1). Here, α and n are constants depending on the foam structure, where the values that have a complex dependence of the foam structure have still not been understood; they depend on whether the pore structure is periodic or disordered (microstructure, unit cell characteristics (open, closed or mixed), pore structure (periodicity or random) and unit cell geometry).

(1) $$E=\alpha E_{0}{\left(\frac{\rho }{\rho_{0}}\right)}^{n}$$

Knudsen [34] and Spriggs [31] proposed an expression to estimate the Young’s modulus as a function of porosity (p) and a parameter that is related to particle stacking (b), Equation (2). This equation is used when foams have low porosity. This expression cannot be used when p = 1 since it does not satisfy the condition that Young’s modulus value must be in so that it is equal to zero.

(2) $$E=E_{0}e^{-bp}$$

Phani and Niyogi [32] proposed Equation (3) to determine the Young’s modulus, where $p_{c}$ is the critical porosity at which E = 0, i.e., the material loses mechanical integrity. The critical porosity depends on the stacking geometry of particles, and the material constant *m* also depends on pore distribution geometry, such as shape and connectivity.

(3) $$E=E_{0}{\left(1-\frac{p}{p_{c}}\right)}^{m}$$

Pabst and Gregorová [30] proposed a model as a function of packing geometry factor (*a*), Equation (5), where *a* is equal to 1 for spherical-shaped pores:

(4) $$E=E_{0}\left(1-ap\right)\left(1-\frac{p}{p_{c}}\right)$$

Nielsen [35] proposed a model to determine Young’s modulus values by considering the porosity and the pore shape factor ($F_{f}$), Equation (5), where $F_{f}$ = 4pA/PE^2^, A is the pore area and PE is the experimental perimeter of the pore.

(5) $$E=E_{0}\left[\frac{{\left(1-p/100\right)}^{2}}{1+\left(1/F_{f}-1)p/100\right)}\right]$$

## 4. Results and Discussion

**Figure 5:**
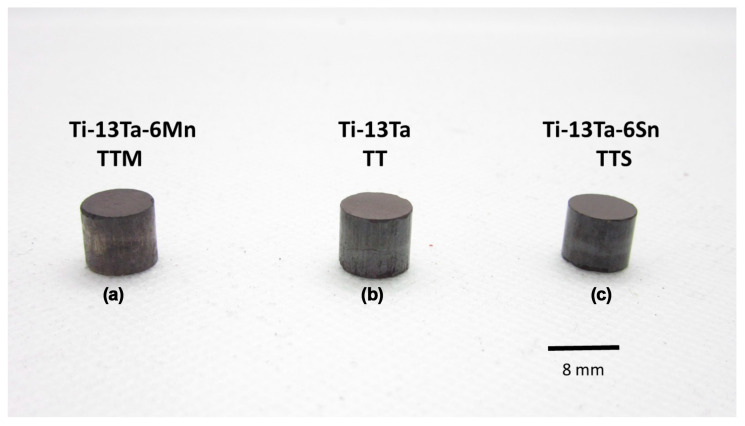
Image of the three consolidated samples, (**a**) Ti-13Ta-6Mn (TTM) (**b**) Ti-13Ta-(TT) and (**c**) Ti-13Ta-6Sn (TTS).

**Figure 6:**
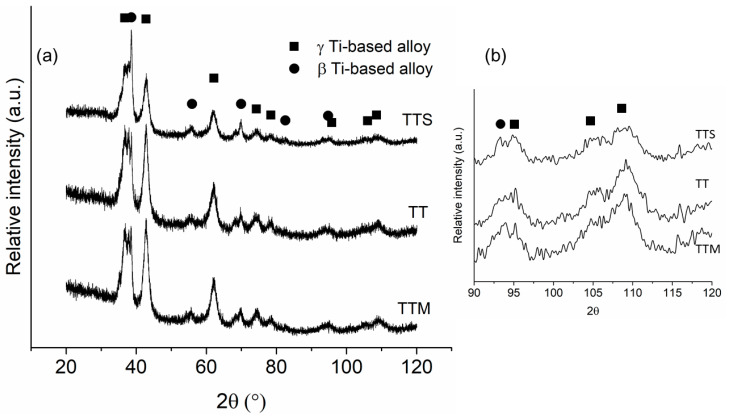
(**a**) XRD patterns of consolidated samples of the Ti-13Ta-6Mn (TTM), Ti-13Ta-(TT) and Ti-13Ta-6Sn (TTS) alloys and (**b**) amplifications of XRD patterns in the 90 to 120 zone.

Cylindrical consolidated samples with dimensions of 8 mm in diameter and 7 mm in height for the TTM, TT and TTS alloys were obtained by a hot-pressing method (Figure 5). The XRD patterns for the consolidated samples showed the presence of two phases, β-phase (BCC crystal structure) and γ-phase (FCC crystal structure) (Figure 6). The reflections of β- and γ-phases exhibited a broadening peak due to the severe plastic deformation promoted during milling [4,70]. Using Rietveld refinements of the XRD patterns, it is possible to observe that the nanocrystalline crystallite size and high microstrain were retained for the γ-phase after consolidation. Both characteristics are required in order for the γ-phase to be present [51,58,59,60]. The outcomes of the refinement process are presented in Table 3. The quality of refinements is assessed using the goodness of fit (GofF) and Rwp indicators, where a refinement is considered excellent when 1 < GofF < 2 and Rwp < 10%. The obtained lattice parameters for the γ Ti-based alloys were near to one another and in accordance with the range previously reported for pure Ti and Ti-based alloys with a γ-phase [49,50,52,59,71,72,73,74,75,76,77,78]. The crystallite size estimated was smaller than 8 nm, which is in agreement with the Gibbs free energy calculation of Xiong et al. [78]. They determined that the fcc phase was stable for nanoparticle sizes smaller than 10 nm. The r.m.s. microstrain (<ε^2^>^1/2^) values were large (between 10^−3^ and 10^2^) indicating the high Young’s energy stored in milled powders. The phase quantification gave γ-phase values between 73 and 78 wt.%, which gave a γ/β phase ratio of around 3/1.

Ti-based alloys with an fcc crystal structure have only begun to be studied recently [56,57,60]. Therefore, there is little information about the mechanical properties of consolidated alloys and foams, as well as the effect that the radial graded porosity has on the Young’s modulus not being known. Considering the latter, the model will be validated for cp-Ti foams, so the results of the RVE-FEA method will be compared with simulated and experimental data of cp-Ti reported in other works.

### 4.1. Validation of Method Using cp-Ti Foams

**Figure 7:**
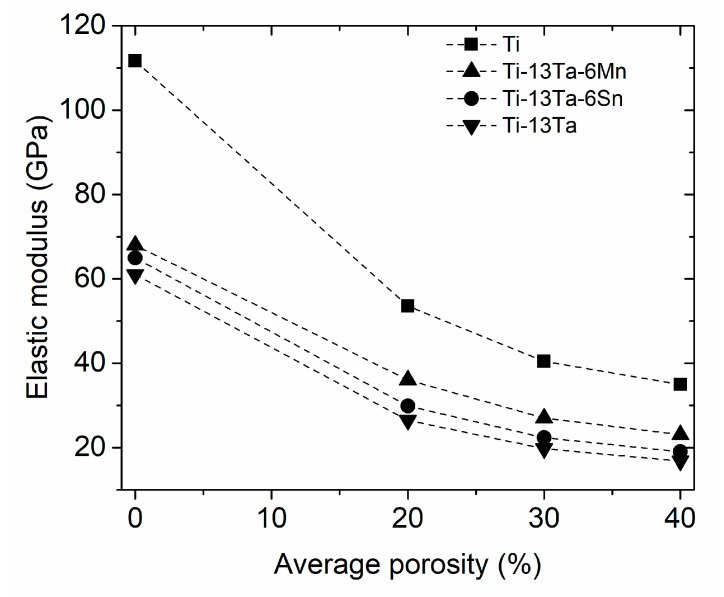
Evolution of Young’s modulus as a function of pore distribution for cp-Ti, Ti-13Ta-6Mn (TTM), Ti-13Ta-(TT) and Ti-13Ta-6Sn (TTS) alloy foams.

**Figure 8:**
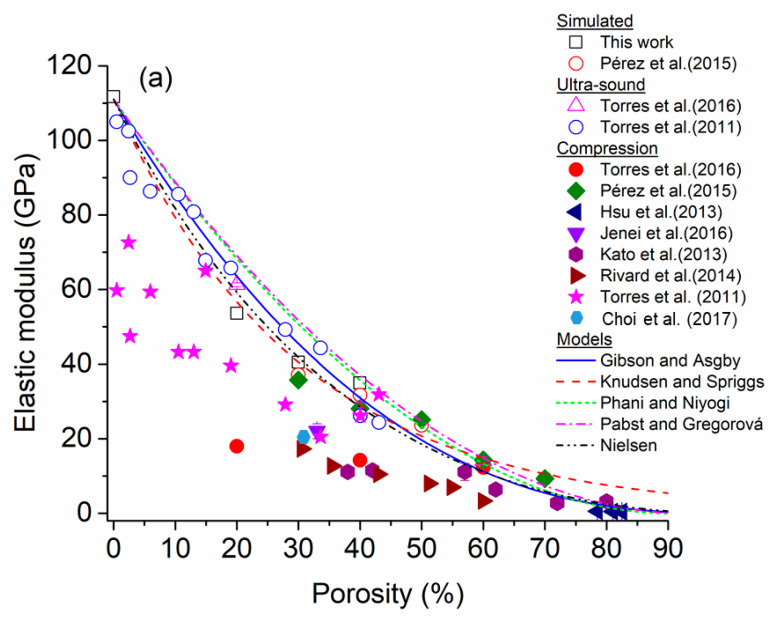

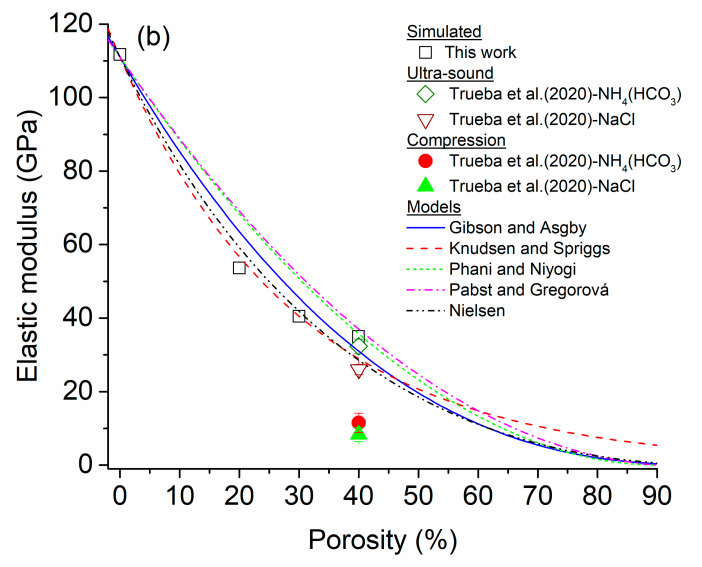
Comparison of Young’s modulus variation as a function of porosity in two types of cp-Ti foams: (**a**) homogeneous porosity [18,39,44,79,80,81,82,85] and (**b**) radial graded porosity [39].

For Ti foams, the E values were determined as a function of graded porosity for the four configurations (Table 1). A diminution of E values was observed from 110 to ~36 GPa for pc-1 and pc-5, respectively (Figure 7). In this figure, the E values for the four Ti alloys foams were added to enable a comparison between all E values. Figure 8a shows a comparison of Young’s modulus values between those simulated and reported in the literature and simulated for Cp-Ti foams with homogeneous porosity [79,80,81,82,83,84]. The following characteristics can be observed: (i) the E values decrease when porosity increases and (ii) the Young’s modulus measured by the ultrasound method (open symbols) gives a larger value than that obtained by mechanical compression (solid symbols). The semi-empirical models (Equations (1)–(5)) were applied using the parameters listed in Table 4 to estimate the E values. However, the calculated Young’s modulus does not undergo accurate representation in the theoretical models, indicating an inadequate capture of the physics behind the homogeneous porosity effect. The semi-empirical models yield larger values than experimental E values, and they are at the upper limit of the data. On the other hand, Figure 8b shows the E values simulated and experimentally measured which have been reported in the literature [39,43] for radial graded porosity. Additionally, the E values determined by the semi-empirical models were included. The experimental E values measured by the ultrasound method of cp-Ti foams [39] give results close to the simulated E values of this research. For this case, the semi-empirical models provide an acceptable correlation between porosity and E values. The Gibson–Ashby model demonstrates a superior fit for the Young’s modulus because it is based on foam’s density, which is an important experimental parameter [83,84,85,86]. Conversely, the remaining models necessitate the measurement or calculation of more intricate parameters, such as b (associated with particle stacking), m (dependent on pore distribution geometry), and Ff (pore shape factor) [4,15,87]. These models have been applied to Ti foams with different amounts of porosity and pore size with acceptable results [4,85,86]. The Young’s modulus results obtained from REV-FEA simulations in this study align well with the findings reported in the literature for foams with radial graded porosity. As a result, the procedure used was considered validated, utilizing a correction factor (β = −0.183) to further enhance the accuracy of the results.

### 4.2. Ti-Based Alloy Foams

**Figure 9:**
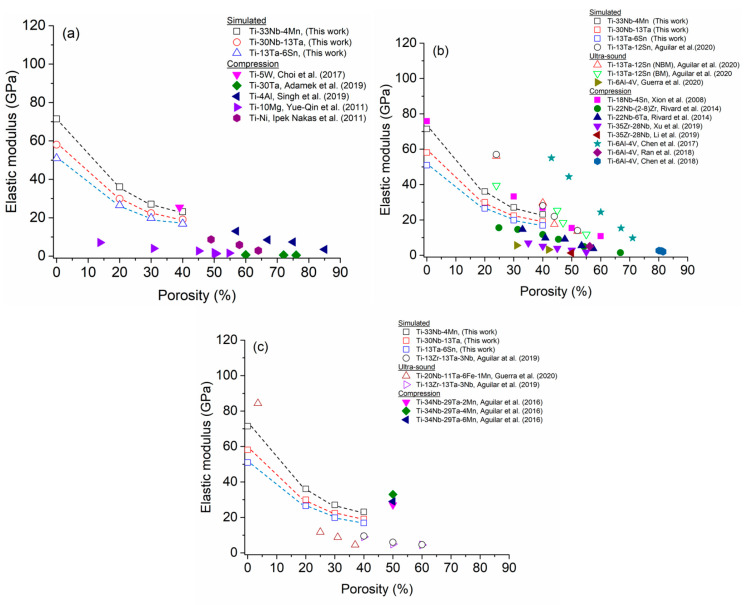
Young’s modulus variation Comparison between Ti-13Ta-6Mn, Ti-13Ta, and Ti-13Ta-6Sn foams and other Ti-based alloy foams with homogeneous porosity; (**a**) binary alloys [17,85,88,89,90] (**b**) ternary alloys and [7,19,20,60,81,94,95,96], and (**c**) multicomponent alloys [4,7,24].

All the Young’s modulus results for Ti-based alloy foams documented in the literature pertain to homogeneous porosity, with none being about radial graded porosity distribution. Therefore, all discussion will be performed considering foams with homogeneous porosity. The E values of the three consolidated Ti alloys with a fcc crystal structure (TTM, TT and TTS) were experientially measured by the ultra-sound method, with their values being ~69, ~61 and ~65 GPa, respectively. Using that information, Young’s modulus values were obtained by simulation according to the method described in Section 3.1. The Young’s modulus of the three Ti alloy foams decreased when porosity levels increased or their porosity average increased (Figure 7). Smaller Young’s modulus values are observed for the radial graded porosity configuration pc-4 (average porosity of 40%), exhibiting a higher porosity average (Table 1). For the TTM, TT and TTS foams, values of ~23, ~19 and ~18 were obtained for the pc-4 configuration (40% porosity), respectively. The Young’s modulus of the three TTM, TTT and TTS foams were compared with data reported by other Ti-based alloy foams (Figure 9). On the other hand, Table 5 provides a literature data summary for the Ti-based alloy foam, such as synthesis method, type of consolidated phase, pore size range, porosity range, methods used to measure and theoretical model parameters used to predict the Young’s modulus. The following discussion will be completed considering the simulation results in this work with data for binary, ternary and multicomponent Ti-based alloy foams that have been reported in the literature.

**(a)**  **Comparison with binary Ti-based alloy foams**

There are a few reports about the Young’s modulus of foams made with binary Ti-based alloys. The main alloying elements used have been W, Ta, Al, Mg and Ni (Figure 9a). The majority of Young’s modulus reported for binary Ti-based alloy foams are for porosity levels higher than 50%. The simulated Young’s modulus (in this work) are higher than all values reported in the literature, and only the value reported by Choi et al. [85] is close to the TTM foam with a 40% porosity (Figure 9a).

The Young’s modulus values of the binary alloy foams were smaller than 30 GPa which is below the upper limit for cortical human bone [80]. In this sense, these foams meet the human bone elasticity requirement. The synthesis process that has been used the most is powder metallurgy that uses NH_4_HCO_3_ particles as a space-holder. The pore size range of almost all foams were within 100–500 μm, making it acceptable to promote the biocompatibility between metallic foam and the human body [37]. The minimal recommendable pore size for bone ingrowth is 100 μm, with larger pore sizes promoting biocompatibility since higher vascularization and oxygenation occur [37]. Choi et al. [85] measured the Young’s modulus values of Ti-5W (wt.%) foam with a 25.4 GPa for a porosity of 39%. Adamek et al. [88] measured the Young’s modulus through compression testing and obtained low values, 0.65, 0.56 and 0.53 GPa for the porosities of 60, 72 and 76%, respectively, for a Ti-13Ta alloy foam. A thermal dealloying method, that added Mg, was used. The macro pore size was between 5 and 90 μm, with smaller pore sizes being between 0.1 and 2 μm. Singh et al. [17] reported a Young’s modulus between 13 and 3.5 GPa for porosity values of 57 and 85%, respectively, in Ti-5Al (wt.%) using (NH_4_)_2_CO_3_ particles as a space-holder, and with the average pore size reported being 225 μm. Ipek Kakaş et al. [89] analyzed the fatigue behavior of Ti-Ni foam using Mg as a space-holder. They measured a Young’s modulus between 8.7 and 2.9 (by compression test) for porosity values ranging from 49 to 64. For Ti-10Mg foams, a Young’s modulus ranging from 7 to 1–5 GPa was reported [90]. For Ti-Zr foams, a Young’s moduli of 15.3 GPa was measured by compression testing for a porosity of 70% [91]. The foam was synthesized by the powder metallurgy method using ammonium hydrogen carbonate particles as the space-holder. The pore size was between 200 and 500 μm.

**(b)**  **Comparison with ternary Ti-based alloy foams**

The majority of recent works about foam correspond to ternary Ti-based alloys. Young’s modulus values as a function of porosity level exhibited a wide variation (Figure 9b). The variation in Young’s modulus for TTM, TT and TTS foams were within a variation range showed by foams values that have been reported in the literature. The wide Young’s modulus values can be explained primarily by the following reason, variations considered include the type and amount of alloying elements, matrix phase type, pore size, shape, and the technique utilized to measure Young’s modulus values. Ti and Ti-based alloys, exhibited two equilibrium phases, α-phase with a hexagonal close-packed (hcp) crystal structure and the β-phase with a body-centered cubic (bcc) crystal structure. The α-phase was present at lower temperatures, while the β-phase at higher temperatures. The α-phase and β-phase exhibited both the highest and lowest Young’s modulus, respectively [60]. For this reason, Ti-based alloys used in biomedical applications are preferable with a β-phase matrix. The mean for the alloying elements that are β-phase stabilizers were Ta, Zr, Nb, V [46], Al as the α-phase stabilizer, and Sn as a neutral element [6]. As mentioned, a metastable face cubic centered (fcc) crystalline structure (γ-phase) has been reported in pure Ti and some Ti-based alloys [49,50,51,52,53,54,55,56], but there is no information about foams with this crystal structure.

Aguilar et al. [92] analyzed the influence that porosity has on the Young’s modulus of Ti-13Ta-12Sn foams synthesized using (NH_4_)_2_CO_3_ as a space-holder, measuring values between 56.1 and 13.4 GPa for a porosity range of 24 to 52%. The values were measured using an ultrasound method. These values were higher than the simulated values for TTS foam; the main difference being that Aguilar et al. produced foams with an irregular pore shape and homogeneous porosity. The simulated Young’s modulus (in this work, open blue square symbol) for Ti-13Ta-6Sn alloy foams were smaller than the Young’s modulus measured in foams coming from the same alloy (open red upper triangle symbol and open green down triangle symbol). This difference between Young’s modulus values exists because of the porosity distribution, radial graded porosity, and homogeneous porosity, respectively. Xiong et al. [7] measured a Young’s modulus variation in Ti-18Nb-4Sn (wt.%) foams between 10.8 and 33.2 GPa for porosities of 60 and 30, respectively. They applied the Gibson–Ashby model and found α and n values of 1 and 2, respectively. Rivard et al. [81] synthesized two Ti-based alloy foams (Ti-22Nb-6Ta (at.%) and Ti-22Nb-(2-8)Zr (at.%)) through the powder metallurgy method while using polymethylmethacrylate as a space-holder, with elastic moduli being measured from compression testing. With Ti-22Nb-6Ta alloy foams, the Young’s modulus was between ~4 and ~15 GPa for the porosities of ~57 and ~33%, respectively, and for the Ti-22Nb-(2-8)Zr alloy foams, 1.5 to 16 GPa were found for porosities of around 67 and 15, respectively. The pore size obtained (D50) for both foam types was between ~100 and 3200 μm (Ti-22Nb-6Ta foam) and 100 and 3100 μm (Ti-22Nb-(2-8)Zr foam). The elastic moduli values for the Ti-35-28Nb foam [93,94] were extremely low, 2.9 and 1.3 for porosities around 50%. For Ti-6Al-4V alloy foams, a Young’s modulus of 7.5, 5.9 and 5.0 GPa for the porosity levels of 44.9, 50.1 and 56.2%, respectively, were measured by a compression test [95]. Guerra et al. [19] reported Young’s modulus values of 5.6 and 3.2 GPa for porosities of 31 and 42%, respectively, that had been measured using an ultrasound method on Ti-6Al-4V (Ti64) foams. The foams were synthesized using (NH_4_)_2_CO_3_ as a space-holder. Other authors have reported lower Young’s modulus for Ti64 foams, such as 2.6 to 2.0 GPa for an amount of porosity of 80.1 to 81.5%, respectively [20]. Foams obtained through the selective laser melting method [20] demonstrated a higher Young’s modulus compared to other foams (Figure 9b). In this case, the pore size and shape influenced the Young’s modulus value. Figure 9b shows that the Young’s modulus values for foams reported in works by Chen et al. [96] follow the same trend as that of the function of porosity (hexagonal and stars symbols).

**(c)**  **Comparison with multicomponent Ti-based alloy foams**

There are few works about multicomponent Ti-based alloy foams focusing on biomedical applications. The simulated Young’s modulus values of TTM, TT and TTS foams are compared with values with three multicomponent Ti-based alloy foams that have been reported in the literature (Figure 9c). The Young’s modulus of TTM, TT and TTS foams are higher than the values of Ti-20Nb-11Ta-6Fe-1Mn and Ti-13Zr-13Ta-3Nb foams and smaller than the values of Ti-34Nb-29Ta-xMn (x = 2, 4 and 6 wt.%). Aguilar et al. [4] studied the effect of Mn content on the Young’s modulus of Ti-34Nb-29Ta-xMn (x = 2, 4 and 6 wt.%). They synthesized the foams using (NH_4_)_2_CO_3_ as a space-holder with 50% of porosity. They measured the elastic moduli by a compression test being performed between 27 and 33 GPa for the amount of Mn. The macro pore size observed was between 100 and 600 μm, with micro pore sizes being smaller than 20 μm. Similar Young’s modulus values for Ti-20Nb-11Ta-16Fe-1Mn (at.%) foam was reported by Guerra et al. [97]. The measured values were obtained by using the ultrasound method with 11.7, 8.8 and 4.5 GPa for porosity values of 25, 31 and 37%, respectively. Meanwhile, Aguilar et al. [13] analyzed the influence that porosity has on the Young’s Modulus of Ti-13Zr-13Ta-3Nb (wt.%) foams. The samples were synthesized using NaCl as a space-holder with porosities of 40, 50 and 60% with Young’s modulus being measured by using the ultra-sound method with 9, 5 and 4.5 GPa, respectively. The macro pore sizes were measured between 100 and 600 μm and with the micropore size being measured with a smaller than 20 μm.

**(d)**  **Brief analysis of obtained results using theoretical models**

Few models are used to calculate the Young’s modulus in foams of multicomponent Ti-based alloys. Table 6 provides the fitted parameter values of semi-empirical models used to estimate the Young’s modulus values of the TTM, TT and TTS foams, in the same way as was performed for cp-Ti foams (Table 4). The Gibson–Ashby (Equation (1)) and Knudsen–Spriggs (Equation (2)) models showed the best results since they take empirical variables such as density and particles stacking, respectively, into consideration. The Gibson–Ashby model has been used more, since density is a physical property that can be easily measured. The α and n fitted values were ranged between 0.90 and 1.23 and 2.49 and 2.57, respectively, with a R-square value of around 0.96. The Gibson–Ashby model gives an acceptable interpretation for the variation in the Young’s modulus as a function of radial graded porosity distribution, seeing that it is based on density. Various works have used parameters α and n equal to 1 and 2, as they produce acceptable results when compared to experimentally measured Ti-5W [85], Ti-18Nb-Sn [7], and Ti-6Al-4V [98]. Chen et al. [20] determined the parameters as α = 1.5 and n = 2, with Aguilar et al. [13] furthermore using the values of α = 0.3 and n = 2.1 for Ti-13Zr-13Ta-3Nb foams. The best fittings for the TTM, TT and TTS foams were obtained when using the Knudsen–Spriggs model (Table 6). The *b* parameter is an empirical constant that varies according to the synthesized method, type of material, and method used to measure the Young’s modulus. This parameter can be varied to obtain a better fitting; therefore, it represents, in a favorable way, the variation in the Young’s modulus as a function of radial graded porosity distribution. For all foams, the *b* values were close to 3. For Ti-30Nb-13Ta foam (obtained by the pulsed electric current sintering technique), the Knudsen–Spriggs model was discussed [57]. The bulk modulus was measured with a compression test at 49 GPa, with parameter *b* being determined to be equal to 3.95, which is larger than b values determined for all three Ti-based alloys (Table 6). On the other hand, Aguilar et al. [13] applied the Knudsen–Spriggs model for Ti-13Zr-13Ta-3Nb foams and determined a *b* value of 6.4. The Phani–Niyogi (Equation (3), Pabst–Gregorová (Equation (4)) and Nielsen (Equation (5)) models depend on porosity values. They provide smaller R-square values along with some inconsistencies in the parameters: (i) the critical porosity values (pc) are different for each foam and model and (ii) *a* and *Ff* parameters in the Pabst–Gregorová and Nielsen models, respectively, must be equal to 1 because the pore shape is a sphere. The last three models do not adequately represent the Young’s modulus change in foams with a radial graded porosity distribution. In general terms, the theoretical models do not fit the experimental data because they do not capture the complexity of the foam physics.

**Table 5:** Literature data summary for Ti-based alloy foam.

Alloy	Synthesis Method	Matrix Phase	Pore Size Range, μm	Pore Shape	Porosity Range, %	Young’s Modulus Range, GPa	Method to Measure Young’s Modulus	Theoretical Model	Ref.
**Binary alloys**									
Ni-Ti (at.%)	Space-holder NaCl		70–400 μm	Blocky	32–36	10–25	CT 0.05 mm/min		[99]
Ti–10Mg (wt.%)	Space-holder NH_4_HCO_3_		100–400 μm	Irregular	13.8–54.8	7.12–1.5	CT 0.5 mm/min		[90]
Ti-51Ni (at.%)	Space-holder Mg	Austenite + quite small MgO	250–600 μm	Equiaxial	49–64	8.7–2.9	CT 0.1 mm/min		[89]
Ti-5Al (wt.%)	Space-holder NH_4_HCO_3_	α-phase	Promedio = 225 μm	Equiaxial	57–85	13–3.5	CT 1 × 10^−2^ s^−1^		[17]
Ti-5W (wt.%)	freeze-cast	β-phase + Widmanstätten α/β structure		Lamellar equiaxed	39	25.4	CT ^(1)^ RT ^(2)^ 1.0 × 10^−3^ s^−1^	G-A α = 1 n = 2	[85]
Ti-Zr (at.%)	Space-holder ammonium hydrogen carbonate		200–500 μm	Irregular	70	15.3	CT RT 1 × 10^−3^ s^−1^		[91]
Ti-13Ta	Dealloying method adding Mg	β-phase	0.1–90 μm	Irregular	60–76	0.65–0.53	CT 1 × 10^−3^ s^−1^		[88]
**Ternary alloys**									
Ti-18Nb-4Sn (wt.%)	Space-holder NH_4_HCO_3_	β-phase	50–450	Irregular	0–60	75.8–10.9	CT 1.0 × 10^−4^ s^−1^	G-A α = 1 n = 2	[7]
Ti-22Nb-6Ta (at.%)	Space-holder pmmc ^(3)^	β-phase and small quantities of α-phase, α’’ + TiC, (Nb,Ti)C + NbC	~100–3200 μm	Irregular	33–57.4	14.6–3.7	CT 2.0 × 10^−3^ s^−1^		[81]
Ti-30Nb-13Ta (at.%)		α-phase + y-phase ^(4)^			0	49	CT 0.05 mm/min	K-S m = 3.95	[57]
Ti-22Nb-(2-8)Zr (at.%)	Space-holder pmmc	β-phase and small quantities of α-phase, α’’ + TiC, (Nb,Ti)C + NbC	100–3100 μm	Irregular	24.8–66.8	15.5–1.5	CT 2.0 × 10^−3^ s^−1^		[81]
Ti-35-Zr-28Nb	Space-holder NH_4_HCO_3_	β-phase	100 to 500 μm	Irregular	35–55	6.9–1.8	CT 2 × 10^−3^ s^−1^		[93,94]
Ti-13Ta-12Sn (at.%)	Space-holder (NH_4_)_2_CO_3_	β-phase with bimodal microstructure	200 to 500 μm	Blocky Irregular	24–55	39.4–11.8	Ultra-sound, transmission technique	G-A α = 0.98 n = 2.9	[92]
Ti-13Ta-12Sn (at.%)	Space-holder (NH_4_)_2_CO_3_	β-phase	200–500 μm	Blocky Irregular	24–52	56.1–13.4	Ultra-sound, transmission technique	G-A α = 1.01 n = 1.96	[92]
Ti-6Al-4V (wt.%)	Space-holder NaCl	α-phase + β-phase	150–250 μm		44.7–70	33–9.5	CT 0.5mm/min	G-A α = 1.0 n = 2.0	[98]
Ti-6Al-4V (wt.%)	Selective laser melting	α-phase + β-phase	559–777 μm (top view) 783–1014 μm (side view)	Cylindrical	43–71	55–9.7	CT RT 1 × 10^−4^ s^−1^	G-A α = 1.5 n = 2.0	[96]
Ti-6Al-4V (wt.%)	Selective laser melting		401–801 μm	Cylindrical	44.9–56.2	7.5–5.0	CT 1.5 mm/min		[95]
Ti-6Al-4V	Space-holder (NH_4_)_2_CO_3_	α-phase + β-phase		Irregular	31–42	5.6–3.2	Ultra-sound, transmission technique		[19]
Ti-6Al-4V	Electron beam melting	α-phase + β-phase	2.5–4 mm	Diamond unit cell	80.1–81.5	2.6–2.0	CT RT 1 × 10^−4^ s^−1^	G-A α = 1.5 n = 2.0	[20]
**Multicomponent alloys**									
Ti-34Nb-29Ta-xMn (x = 2, 4 and 6 wt.%).	Space-holder NH_4_HCO_3_	α-phase + β-phase + TiO	100–600 μm <20 μm	Irregular	50	27–33	CT 0.125 mm/min	K-N b = 3.36 P-N m = 1.93 pc = 83% P-G a = 1 Pc = 83% G-A α = 1 n = 2 N Ff = 0.7	[4]
Ti-20Nb-11Ta-16Fe-1Mn (at.%)	Space-holder NH_4_HCO_3_	α-phase + β-phase	10 μm	Irregular	25–37	11.7–4.5	Ultra-sound, transmission technique		[97]
Ti-20Nb-11Ta-16Fe-1Mn (at.%)	Arc-melting	α-phase + β-phase			3.5	84.3	Ultra-sound, transmission technique		[97]
Ti-13Zr-13Ta-3Nb (wt.%)	Space-holder NaCl	β-phase	100 to 600 μm	Equiaxial–Irregular	40–60	9–4.5	Ultra-sound, transmission technique	K-N b = 6.4 G-A α = 0.5 n = 2.1	[13]

^(1)^ Compression test, ^(2)^ room temperature, ^(3)^ pmmc = polymethylmethacrylate, ^(4)^ γ is a fcc crystal structure.

## 5. Conclusions

The Ansys Material Designer module is capable of creating foam models with a radial graded porosity distribution. However, the values that were achieved differ from the experimental measurements for cp-Ti foams, since the program does not consider the intrinsic anisotropy properties produced by the pore distribution. The problem was solved by introducing a correction factor (β) which, in this work, had a value of −0.183. This methodology has the potential to simulate mechanical properties of foams with different configurations that can be approximated considering, type of material, porosity, pore size and pore distribution. The model and simulation restrictions were validated with experimental data for cp-Ti foams since they have only been extensively studied in the last 15 years. The decrease in simulated Young’s modulus as a function of porosity for foams with radial graded porosity aligns more closely with the experimental values reported in the literature.

In the case of the three TTM, TT and TTS foams, the simulated Young’s modulus decreased when porosity increased, with their values being within the range required for different human bones. For the configurations pc-3 and pc-4 (30 and 40% porosity), the simulated values were between 23 and 27, 19 and 22 and 17and 20 GPa for the TTM, TT and TTS foams, respectively. Therefore, the foams with Ti-based alloy with γ/β phase ratio exhibited good potential to be used as a biomaterial. Despite this, more investigation is required to explore certain properties such as strength, fatigue, biocompatibility, etc.

The application of simple models to estimate the simulated Young’s modulus resulted in significant discrepancies. Models based on physical properties, particularly density, yielded more accurate outcomes compared to those using other parameters. In this context, the Gibson–Ashby and Knudsen–Spriggs models can be employed as an initial approach to estimate the effect of the radial graded porosity distribution on the Young’s modulus.

## Figures and Tables

**Table 1:** Radial graded porosity distribution configurations used in simulations.

	Porosity (*v*/*v*%)			
Radial Graded Porosity Configuration (pc)	Core	Inner Shell	Outer Shell	Average Porosity (ap)
pc-1	CS	CS	CS	0
pc-2	CS	20	40	20
pc-3	CS	30	60	30
pc-4	20	40	60	40

CS: Consolidated sample.

**Table 2:** cp-Ti and Ti-based alloy mechanical parameters used.

Material	Young’s Modulus, GPa	Poisson Coefficient	Density, g/m^3^
cp-Ti (grade 4)	110	0.31	4.51
Ti-13Ta-6Mn (TTM)	69	0.31	5.96
Ti-13Ta-(TT)	61	0.31	7.30
Ti-13Ta-6Sn (TTS)	65	0.31	6.24

**Table 3:** Microstructural parameters obtained from Rietveld refinements using MAUD software for the γ-phase.

Alloy	Lattice Parameter, nm	Crystallite Size, nm	Microstrain, <ε^2^>^1/2^	Amount, wt.%	GofF	Rwp
TTM	0.42292	7.6	6.9 × 10^−3^	76	1.0	8.8
TT	0.42247	7.3	5.8 × 10^−3^	77.8	1.0	10.0
TTS	0.42302	7.0	7.6 × 10^−3^	73.5	1.1	10.5

**Table 4:** Parameter employed in semi-empirical models for estimating Young’s modulus values.

Material	Properties	Gibson and Ashby	Knudsen and Spriggs	Phani and Niyogi	Pabst and Gregorová	Nielsen
cp-Ti	E = 111 GPa d = 4.50 g/cc	α = 1 n = 2.5	b = 3.36	m = 1.93 pc = 90%	a = 1 pc = 90%	Ff = 0.5

**Table 6:** Fitted parameters values of models to estimate Young’s modulus values of the Ti-13Ta-4Mn, Ti-13Ta and Ti-13Ta-6Sn alloy foams.

Alloy	Properties	Gibson–Ashby	Knudsen–Spriggs	Phani–Niyogi	Pabst–Gregorová	Nielsen
Ti-13Ta-6Mn	E = 70 GPa d = 5.96 g/cc	R-square = 0.96 α = 1.23 n = 2.57	R-square = 0.99 b = 3.06	R-square = 0.56 m = 1.45 pc = 90%	R-square = 0.94 a = 1.1 pc = 78%	R-square = 0.98 Ff = 0.52
Ti-13Ta	E = 57 GPa d = 7.30 g/cc	R-square = 0.96 α = 1.0 n = 2.51	R-square = 0.99 b = 3.01	R-square = 0.89 m = 2.05 pc = 90%	R-square = 0.95 a = 1.1 pc = 82%	R-square = 0.98 Ff = 0.58
Ti-13Ta-6Sn	E = 51 GPa d = 6.24 g/cc	R-square = 0.97 α = 0.98 n = 2.49	R-square = 0.99 b = 3.05	R-square = 0.90 m = 2.46 pc = 90%	R-square = 0.95 a = 1.1 pc = 81%	R-square = 0.98 Ff = 0.56

## Data Availability

The raw/processed data required to reproduce these findings can’t be shared at this time due to technical or time limitations.
